# Recombinant expression and immunogenicity verification of Dabie bandavirus proteins Gn and Gc

**DOI:** 10.3389/fmicb.2025.1651194

**Published:** 2025-09-05

**Authors:** Xiuyu Lou, Haiyan Mao, Yihan Lou, Yi Sun, Feng Wang, Minjie Wang, Lijun Zhang, Zhihan Fang, Hao Yan, Huijun Zhang

**Affiliations:** ^1^Zhejiang Key Laboratory of Public Health Detection and Pathogenesis Research, Department of Microbiology, Zhejiang Provincial Center for Disease Control and Prevention, Hangzhou, China; ^2^Department of Clinical Laboratory, National Cancer Center/National Clinical Research Center for Cancer, Cancer Hospital, Chinese Academy of Medical Sciences and Peking Union Medical College, Beijing, China; ^3^Minhang Hospital, Fudan University, Shanghai, China; ^4^Yangpu Hospital, Tongji University, Shanghai, China; ^5^Institute of Laboratory Animal Sciences, Chinese Academy of Medical Sciences and Peking Union Medical College, Beijing, China

**Keywords:** Dabie bandavirus, envelope protein Gn, envelope protein Gc, prokaryotic expression, immunogenicity

## Abstract

**Introduction:**

Dabie bandavirus (DBV), a newly identified pathogen transmitted to humans via ticks bites, is the etiologic agent of severe fever with thrombocytopenia syndrome (SFTS). This disease is associated with a high mortality rate and constitutes a substantial threat to global public health. This study aimed to recombinantly express and characterize the immunogenicity and antigenicity of the DBV envelope proteins Gn and Gc.

**Methods:**

The recombinant plasmids pET15b-Gn and pET15b-Gc were constructed and expressed in *E.coil*. The expressed proteins were purified, and rabbit polyclonal antibodies (anti-rGn-IgG and anti-rGc-IgG) were prepared through a four-immunization regimen. The antigenic specificity of the recombinant proteins was assessed, and their performance was evaluated for detecting DBV IgM antibodies in samples.

**Results:**

The rGn and rGc proteins were successfully expressed and purified, exhibiting molecular weights consistent with theoretical predictions. Antibody titers in immunized rabbits reached 1:512,000 for anti-rGn-IgG and 1:256,000 for anti-rGc-IgG. The proteins showed no significant cross-reactivity with other prevalent arboviruses. When used as antigens in assays, the rGn- and rGc-coated plates detected DBV IgM antibodies in 84.21% and 89.47% of positive samples, respectively.

**Discussion:**

This study confirmed that the prokaryotically expressed DBV Gn and Gc proteins possess favorable immunogenicity, addressing the critical knowledge gap in evaluating of the immunogenic efficacy of prokaryotically expressed Gn and Gc. Currently, research on DBV’s pathogenic mechanisms, protein structure, and functions remains limited, and this findings provide a foundation for the development of DBV-related vaccines and drugs.

## 1 Introduction

The Dabie bandavirus (DBV), formerly known as Severe Fever with Thrombocytopenia Syndrome Virus (SFTSV) until 2022, is a segmented negative-sense RNA virus belonging to the genus *Phlebovirus* (family *Phenuiviridae*, order *Bunyavirales*). Transmitted to humans primarily through tick bites, DBV causes severe fever with thrombocytopenia syndrome (SFTS) (Zhang et al., 2025). Infection can lead to fever, thrombocytopenia, and hepatic and renal dysfunction, and may rapidly progress to death due to disseminated intravascular coagulation, shock, and respiratory failure, with a mortality rate as high as 30% (Wan et al., 2025). DBV predominantly circulates between wild animals and livestock via the longhorned tick, infecting humans through tick bites during outdoor activities (Casel et al., 2021). With geographical spread, ecological changes, climate warming, and population movement, DBV has emerged as a global public health threat (Chen et al., 2025; Saijo, 2022; Zhang et al., 2024).

Current research on DBV mainly focuses on epidemiology and molecular detection techniques, with relatively limited exploration of its pathogenic mechanisms and virological characteristics (Lei et al., 2025; Shu et al., 2025). The DBV genome comprises three single-stranded negative-sense RNA segments: L, M, and S. The L segment encodes RNA-dependent RNA polymerase, the M segment encodes the precursor proteins that are processed into the envelope proteins Gn and Gc, and the S segment encodes the nucleoprotein (NP) and nonstructural protein (NS) (Li, 2015). As major surface antigens of the virus, envelope proteins Gn and Gc are key mediators of DBV adhesion to platelets or host cells, playing important roles in viral attachment, entry into host cells, and induction of platelet damage. They also represent key targets for vaccine development and serological diagnosis. Structural biology studies have identified conserved linear epitopes within the receptor-binding domain (RBD) of the Gn protein (Zhang et al., 2025), suggesting that the prokaryotic expression system may preserve core antigenic domain to achieve high immunogenicity. However, systematic evaluation of the immunogenic efficacy of prokaryotically expressed Gn remains lacking.

In this study, genetic engineering techniques were applied for recombinant expression of DBV envelope proteins Gn and Gc. High-purity recombinant proteins were obtained using affinity chromatography, and animal experiments were conducted to verify their immunogenicity and antigenicity. These efforts lay the groundwork for further in-depth studies on the structure and function of the DBV Gn and Gc proteins and for precisely locating their antigenic epitopes, and provide robust support for the subsequent development of DBV-specific subunit vaccines and neutralizing antibody detection methods.

## 2 Materials and methods

### 2.1 Materials

Vero cells (laboratory stock), specific primers (Sangon Biotech, Shanghai), RNA extraction kit (RNeasy Mini Kit, Qiagen), reverse transcription kit (RevertAid III First Strand cDNA Synthesis Kit, Thermo Fisher Scientific), amplification reagents (Platinum@Taq DNA Polymerase High Fidelity Kit, Life), gel extraction kit (Gel Extraction kit, Omega), BL21(DE3) cells (Sangon Biotech, Shanghai), restriction endonuclease *NdeI* and *XhoI* (TaKaRa), T4 DNA ligase (Beijing TransGen Biotech), Ni-NTA affinity column (Beijing TransGen Biotech), rabbit anti-6His antibody (Beijing TransGen Biotech), HRP-conjugated goat anti-rabbit antibody (Sigma), BCA protein quantification kit (Beijing TransGen Biotech).

### 2.2 Methods

#### 2.2.1 DBV cultivation and purification

Vero cells (ATCC Number: CCL-81) were resuscitated and propagated to passage 8, cultured in minimal essential medium (MEM) supplemented with 10% fetal bovine serum (FBS), and incubated at 37°C with 5% CO_2_ until reaching 90% confluence.

DBV virus (Zhejiang/01/2011(SFTSV), GenBank accession: KJ597824.1), isolated from a Zhejiang SFTS patient and stored in our laboratory, was inoculated into monolayer Vero cells in MEM maintenance medium (2% FBS) and incubated at 37°C with 5% CO₂. Cells were monitored daily for cytopathic effects (CPE). Upon observation of 80–100% CPE, viral supernatant were collected and concentrated by ultracentrifugation over a 20% sucrose cushion at 32,000 rpm for 4 h at 4°C. Purified DBV particles were stored at −70°C.

#### 2.2.2 Primer design and synthesis

Sequence analysis of multiple DBV strains from GenBank revealed >99% amino acid sequence similarity in envelope glycoproteins Gn and Gc across different strains, indicating remarkable evolutionary conservation of these structural proteins. This high conservation supports the utility of any representative strain for Gn/Gc protein characterization. Based on these findings, two pairs of specific primers targeting the complete Gn and Gc gene sequences of the Zhejiang/01/2011 (SFTSV) reference strain were designed.

Gn-F (5′-GACACGACACCATATGGACTCAGGCCC-3′), Gn-R (5′-GTGTCCTCGAGTCATTATCCATAG-3′), amplifying 1–900 bp of the Gn gene; Gc-F (5′-GACACGACACCATATGTGTGACGA-3′) and Gc-R (5′-GTGTCCTCGAGTCATTAGCGCACGATATACGA-3′), amplifying 1–1,200 bp of the Gc gene. Restriction enzyme sites for *NdeI* and *XhoI* were incorporated into the primers (underlined), with flanking sequences including GACACGACAC, GTGTC, and TCATTA.

#### 2.2.3 RNA extraction, reverse transcription and PCR amplification

Total RNA was extracted from DBV using an RNA extraction kit, and cDNA was synthesized using a reverse transcription kit. The DBV Gn and Gc genes were amplified from this cDNA via PCR under the following conditions: 95°C for 2 min; 35 cycles of 94°C for 30 s, 55°C for 30 s, and 68°C for 1 min; followed by a final extension at 68°C for 10 min, according to the PCR kit manufacturer’s instructions. The resulting PCR products were analysed by 1.5% agarose gel electrophoresis and purified using a gel extraction kit.

#### 2.2.4 Construction of recombinant plasmids

The purified PCR products and the expression vector pET15b were double-digested with *NdeI* and *XhoI*. The digested products were purified using a gel extraction kit and ligated with T4 DNA ligase. The ligation products were transformed into competent BL21(DE3) cells, and single colonies were selected for plasmid extraction and PCR verification. Positive recombinant plasmids were sequenced and named pET15b-Gn and pET15b-Gc.

#### 2.2.5 Expression and purification of recombinant proteins

Recombinant plasmids pET15b-Gn and pET15b-Gc were expressed in *E. coli*. Colonies were cultured in ampicillin-containing LB broth at 37°C until optical density (OD) reached 0.6, IPTG was added, and the culture was transferred to 16°C for induction of expression. Cells were harvested, sonicated, and centrifuged. The pellet containing inclusion bodies was solubilized, and His-tagged rGn and rGc were purified by Ni-NTA affinity chromatography. Proteins concentration were quantified using a BCA kit. For SDS-PAGE, samples (1 μg/lane) were resolved on 12% gels (5% stacking gel) with Tris-Glycine buffer at 80 V for 30 min and 120 V for 60 min, followed by Coomassie Blue staining for 20 min and destaining. Western blot analysis used the same electrophoretic conditions. Proteins were wet-transferred to PVDF membranes at 250 mA for 90 min. After blocking with 5% skim milk at 37°C for 2 h, the membranes were incubated with rabbit anti-His tag antibody and HRP-conjugated goat anti-rabbit IgG at 37°C for 1 h. Bands were visualized using a TMB substrate.

#### 2.2.6 Preparation of rabbit anti-rGn-IgG and anti-rGc-IgG antibodies

Healthy female New Zealand white rabbits (4 months old, 2 kg) were used, with 4 rabbits per group. The initial immunization dose was 0.3 mg per rabbit, and the subsequent immunizations (second, third, and fourth) were 0.15 mg per rabbit. The antigen for the initial immunization was emulsified with an equal volume of Freund’s complete adjuvant, while the antigens for the subsequent immunizations were emulsified with an equal volume of Freund’s incomplete adjuvant. The rabbits were immunized once a week for a total of 4 weeks. After the final immunization, blood was collected from the rabbits, and serum was separated. Pre-immune sera were collected as a negative control before the initial immunization. IgG was purified from the serum using ammonium sulfate precipitation and DEAE-52 ion exchange chromatography (1.6 × 20 cm), which was conducted by Shanghai Sangong Biological Co., Ltd.

#### 2.2.7 Indirect ELISA for determination of antibody titers against rGn and rGc

The proteins were diluted to a final concentration of 10 μg/mL in 0.05 mol/L carbonate buffer (pH 9.6) and coated onto 96-well plates at 100 μL per well, incubated overnight at 4°C. The next day, the plates were washed three times with PBST (0.01 M PBS with 0.05% Tween-20) for 3 min each, and then the wells were patted dry. The wells were blocked with 100 μL of PBST containing 10% fetal bovine serum at 37°C for 60 min. After three 3-min washes with PBST, serially diluted antibodies (initial dilution 1:1000) were added and incubated at 37°C for 1 h. Following three 3-min PBST washes, HRP-conjugated goat anti-rabbit IgG (1:8000) was incubated at 37°C for 40 min. After five 3-min PBST washes, 100 μL of substrate solution (TMB) was added and incubated in the dark for 15 min. The reaction was terminated with 50 μL of 2 mol/L H_2_SO_4_, and the OD_450_ was measured. A result was designated as positive if the mean OD_450_ value of the diluted serum exceeds the mean value of the negative control by a factor of 2.5; otherwise, it was designated as negative.

#### 2.2.8 Western blot analysis for antibody identification

The supernatant of DBV-infected cell cultures was sonicated, and the proteins were separated by SDS-PAGE and then transferred to a PVDF membrane using a semi-dry blotting apparatus. The membrane was blocked with 5% skim milk for 2 h, washed, and then incubated with primary antibodies (rabbit anti-rGn-IgG and anti-rGc-IgG diluted 1:500) for 2 h with gentle shaking. After washing, the membrane was incubated with a secondary antibody (HRP-conjugated goat anti-rabbit antibody diluted 1:8000) for 2 h with gentle shaking. The membrane was then washed and developed with TMB substrate in a darkroom.

#### 2.2.9 Antigenicity assay of recombinant proteins

The recombinant proteins rGn, rGc, and BSA (negative control) were diluted to 10 μg/mL in 0.05 mol/L carbonate buffer (pH 9.6), coated onto 96-well plates (100 μL/ well), and incubated overnight at 4°C. The next day, the plates were washed three times with PBST for 3 min each and patted dry. Each well was blocked with 100 μl PBST containing 10% fetal bovine serum and incubated at 37°C for 60 min. After washing three times with PBST (0.05% Tween-20) for 3 min each and patted dry, the plates were stored at 4°C.

Fifteen clinical serum samples (five groups) were selected from the infectious disease recheck samples of Zhejiang Provincial Centers for Disease Control and Prevention, including three DBV IgM antibody-positive samples, three dengue virus IgM antibody-positive samples, three Chikungunya virus antibody-positive samples, three Japanese encephalitis virus IgM antibody-positive samples, and three normal human serum samples. Each experiment was performed in thriplicate. All serum samples were diluted 1:10 and used as primary antibodies, incubated at 37°C for 1 h. The plates were then washed five times with PBST for 3 min each. HRP-conjugated goat anti-rabbit IgG was diluted 1:8000 and incubated at 37°C for 40 min. After washing five times with PBST for 3 min each, 100 μL of substrate solution (TMB) was added to each well and reacted in the dark for 15 min. The reaction was terminated by adding 50 μL of 2 mol/L sulfuric acid, and the OD values were measured at a wavelength of 450 nm using a microplate reader.

Nineteen case samples for DBV IgM antibody-positive were selected and tested using microplates coated with rGn and rGc proteins to calculate the positive detection rates of rGn and rGc. The detected results were analysed by GraphPad Prism 7.0.

## 3 Results

### 3.1 Induction of recombinant plasmids in *E. coli*

**Figure 1:**
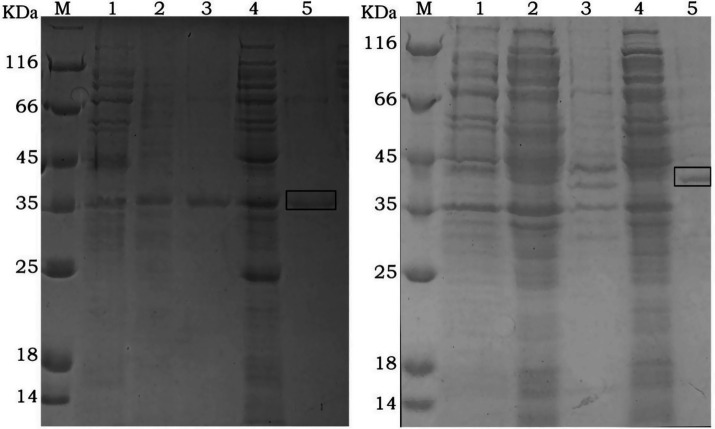
SDS-PAGE analysis of recombinant rGn (Left) and rGc (Right) protein expression M: protein marker; 1: Total cellular proteins before IPTG induction; 2: Soluble fraction (supernatant) after induction with 0.5 mM IPTG; 3: Insoluble fraction (pellet) after induction with 0.5 mM IPTG; 4: Soluble fraction (supernatant) after induction with 1.0 mM IPTG; 5: Insoluble fraction (pellet) after induction with 1.0 mM IPTG. The boxed area indicates the size of the target protein.

The recombinant plasmids pET15b-Gn and pET15b-Gc were induced in *E. coli* BL21(DE3) with 0.5 mM and 1 mM IPTG at 37°C for 4 h. After induction, SDS-PAGE analysis revealed distinct protein expression bands for both constructs. The optimal IPTG concentration was determined to be 1 mM. For the rGn construct, the target protein was detected in both the supernatant and the pellet, indicating that inclusion bodies could be dissolved and purified together. The molecular weights of the recombinant proteins rGn and rGc were approximately 35 kDa and 43 kDa, respectively, which were consistent with the expected theoretical values (Figure 1).

### 3.2 Purification and Western blot identification of recombinant proteins

**Figure 2:**
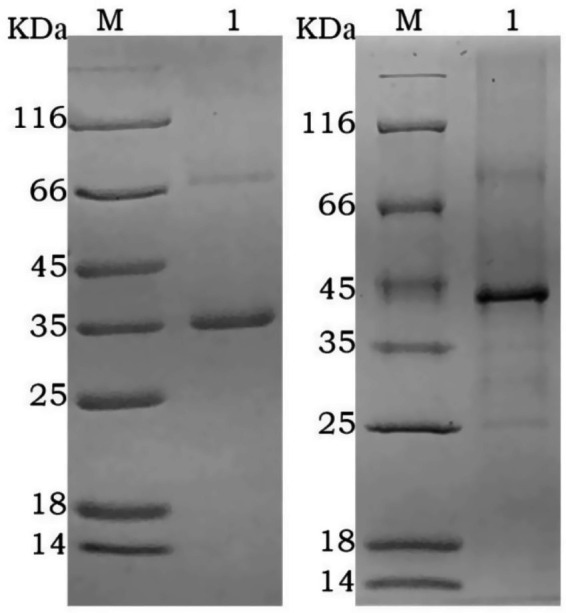
SDS-PAGE analysis of purified recombinant protein M: protein marker; 1: rGn; 2: rGc.

**Figure 3:**
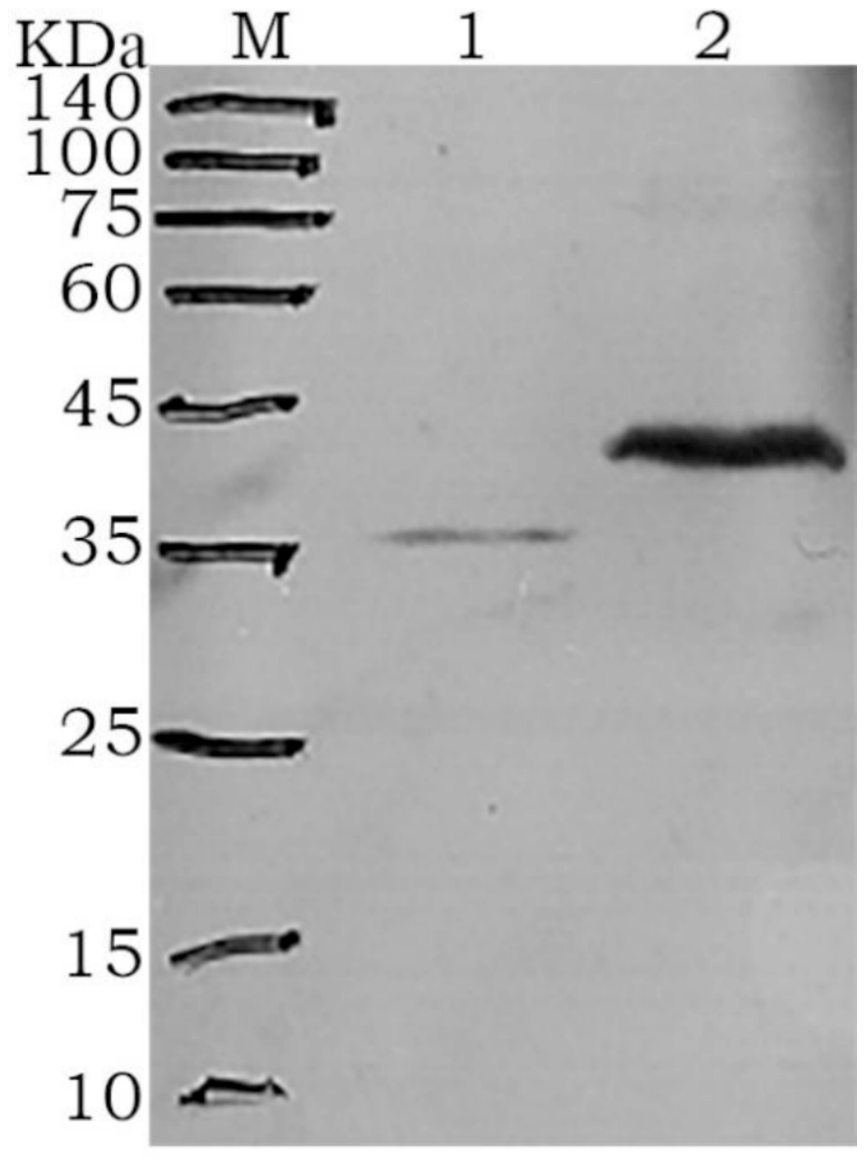
Western blot analysis of the fusion proteins M: protein marker; 1: rGn; 2: rGc.

**Figure 4:**
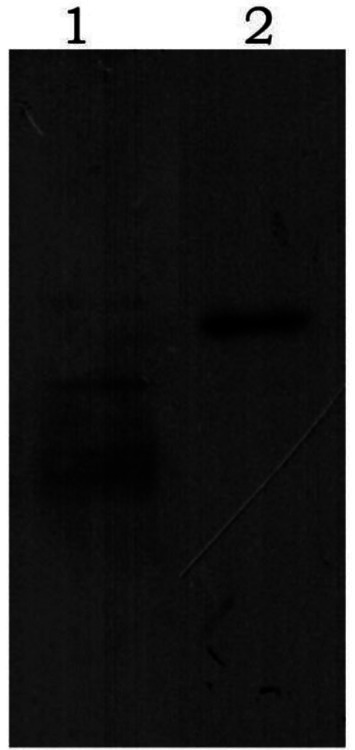
Western blot analysis of purified antibody binding to DBV 1: rGn-IgG immunobinding; 2: rGc-IgG immunobinding.

After purification using a Ni-NTA affinity column, SDS-PAGE analysis showed that the target proteins were obtained with high purity (Figure 2). The recombinant proteins were specifically recognized by anti-6His antibodies, with visible fusion proteins of the expected size, confirming the expression of rGn and rGc proteins in *E. coli* (Figure 3). A total of 16 mL of rGn protein was obtained and aliquoted 1 mL per tube, with a concentration of 0.4 mg/mL (total 6.4 mg). The rGc protein was obtained in a total volume of 10 mL, aliquoted into 1 mL per tube, with a concentration of 0.6 mg/mL, yielding a total protein amount of 6 mg. Western blot analysis showed that the rabbit anti-rGn-IgG and anti-rGc-IgG antibodies effectively bound to the DBV (Figure 4).

### 3.3 Determination of serum antibody titers

One week after the fourth immunization, blood was collected from the animals, and the serum was diluted. The purified antibodies rGn-IgG and rGc-IgG were diluted 1:1000 and further serially diluted up to 512,000 times. Indirect ELISA was used to determine the antibody titers in the antiserum, with a positive result defined as an OD value ratio of the dilution group to the negative control group ≥2.5. The results showed that after four immunizations in New Zealand white rabbits, the titer of rGn-IgG antibodies reached 1:512,000, and the titer of rGc-IgG antibodies reached 1:256,000, indicating that the Gn and Gc proteins expressed in the prokaryotic system had good immunogenicity (Table 1).

**Table 1:** Titers of purified rabbit anti-rGn IgG and anti-rGc IgG antibodies.

Dilution factor (1:)	rGn-IgG (*n* = 4)			rGc-IgG (*n* = 4)		
	Mean OD	Dilution group OD/Negative Control group OD	Result	Mean OD	Dilution group OD/Negative Control group OD	Result
1,000	2.060	36.300	+	1.381	40.625	+
2,000	1.832	32.140	+	1.316	38.691	+
4,000	1.728	30.311	+	1.202	35.353	+
8,000	1.492	26.180	+	1.036	30.478	+
16,000	1.302	22.838	+	0.840	24.713	+
32,000	0.952	16.702	+	0.613	18.037	+
64,000	0.830	14.561	+	0.399	11.721	+
128,000	0.578	10.145	+	0.245	7.191	+
256,000	0.281	4.925	+	0.142	4.169	+
512,000	0.155	2.719	+	0.070	2.066	−
Mean OD of Negative Control group	0.057			0.034		

“+“means Positive, “−” means Negative.

### 3.4 Evaluation of recombinant protein antigenicity and diagnostic applications

The recombinant proteins were used to coat microplates, and five groups of serum samples were tested to measure the OD values. One-way ANOVA (assuming equal mean OD values for the five serum groups, i.e., H0, and unequal values for at least two groups, i.e., H1) and a *post hoc* test (Tukey HSD) were used to evaluated the specificity and cross-reactivity of the proteins. The results showed that both proteins exhibited strong reactivity to DBV (OD > 1.3), with significant differences observed between the groups for both rGn (*F* = 3231.93, *p* < 0.05) and rGc (*F* = 1845.6, *p* < 0.05) coated plates (Table 2). rGn exhibited excellent specificity in terms of cross-reactivity; it showed no significant cross-reactivity in the DENV/CHIKV/JEV group (no difference from NC), whereas rGc showed significant cross-reactivity, with significantly higher OD values than NC in both the CHIKV and JEV groups (*p* < 0.05).

**Table 2:** OD values of five groups of serum samples.

Type	rGn-IgM (*n* = 3)			rGc-IgM (*n* = 3)		
	OD	*F*	*p*	OD	*F*	*p*
DBV	1.325 ± 0.037	3231.93	<0.05	1.415 ± 0.043	1845.6	<0.05
DENV	0.068 ± 0.004			0.050 ± 0.003		
CHIKV	0.057 ± 0.005			0.112 ± 0.009		
JEV	0.114 ± 0.008			0.132 ± 0.009		
NC	0.016 ± 0.004			0.022 ± 0.004		

The rGn-coated microplate detected 16 positive samples out of 19 DBV IgM antibody-positive samples, with a detection rate of 84.21% (16/19). The rGc-coated microplate detected 17 positive samples out of 19, with a detection rate of 89.47% (17/19), including the 16 positive samples detected by the rGn-coated plate. McNemar’s test showed *p* = 1.0 (> 0.05), with a 95% confidence interval of (−0.0505, 0.1557). There was no statistically significant difference in the positivity rates between rGn-coated microplates and rGc-coated microplates. Cohen’s kappa analysis revealed an observational concordance of 94.74% (18/19), with a kappa coefficient of 0.771, indicating good agreement between the two methods (kappa coefficient: <0.4 = poor, 0.4–0.6 = moderatet, 0.6–0.8 = good, >0.8 = excellent).

## 4 Discussion

Since its first identification in 2009, DBV infections have been reported across multiple regions in China, including Shandong, Hunan, Zhejiang, and Henan provinces (Dong et al., 2025; Liu et al., 2024; Yao et al., 2019; Lei et al., 2025). Subsequently, tick-borne human cases have been documented in the United States, Japan, and South Korea, Thailand, Vietnam and Pakistam, confirming its emerging global distribution (Silvas and Aguilar, 2017; Kobayashi et al., 2020; Kwon et al., 2024; Rattanakomol et al., 2023; Tran et al., 2022; Zohaib et al., 2020). DBV infection is characterized by an acute onset, rapid progression, and a high mortality. Currently, no specific antiviral therapies exist, with treatment relying primarily on broad-spectrum antiviral agents for symptomatic relief. This approach often results in prolonged treatment cycles and uncertain outcomes, significantly compromising patient quality of life.

As a member of the *Bunyaviridae* family, DBV possesses conserved envelope proteins Gn and Gc, which are taxonomic hallmarks of this group (Liu et al., 2024). The ectodomains of these proteins serve as primary targets for neutralizing antibodies directed against linear epitopes, consistent with other bandaviruses (Överby et al., 2008; Lee et al., 2023). Gn critially mediates viral attachment and cellular entry, contributing significantly to pathogenicity (Jo et al., 2019).

This study demonstrates a significant methodological advance through the successful prokaryotic expression of immunogenic extracellular domains of Gn (1–900 bp) and Gc (1–1,200 bp). While earlier work achieved prokaryotic expression of Gn/Gc fragments (Zhang et al., 2013), we provide the evidence that prokaryotically expressed ectodomain segments (rGn 1-300aa, rGc 1-400aa) retain potent immunogenicity. This achievement addresses well-documented challenges associated with eukaryotic expression systems, including technical complexity (Teresa et al., 2016) and glycosylation-induced instability (Xu et al., 2022).

ELISA data establish the diagnostic utility of these antigens for acute-phase detection. Plates coated with rGn or rGc exhibited 85% sensitivity for IgM detection in clinically confirmed acute-phase sera, with minimal cross-reactivity against dengue virus, Japanese encephalitis virus (JEV), or chikungunya virus. This IgM-specific reactivity provides a foundation for developing rapid DBV diagnostic assays in regions with co-circulating arboviruses. The prioritization of IgM validation aligns with the study’s focus on acute infection diagnosis, although limitations include a constrained sample size of acute-phase sera and the need for broader clinical validation. These immunogenicity findings address persistent questions in the field. Unlike studies that did not assess immunogenicity (Zhou et al., 2015) or lacked mechanistic insights (Han et al., 2024), our data definitively demonstrate IgM-specific antibody induction by prokaryotic ectodomain antigens. Furthermore, while prior work expressed Gn without functional validation (Fu et al., 2018), our approach yields purified ectodomain antigens suitable for standardized evaluation.

Methodologically, our strategy offers distinct advantages. Chemical synthesis enabled efficient production of genes encoding immunodominant ectodomains, while the prokaryotic system generated soluble, non-glycosylated proteins, overcoming the cost and stability limitations inherent in eukaryotic expression (Xu et al., 2022). This establishes a scalable platform for diagnostic antigen production. Collectively, these findings validate prokaryotic expression of ectodomain antigens for DBV diagnostics. The 85% IgM detection rate supports two key applications: the development of IgM-based rapid diagnostic tests and the provision of stable antigens for epitope-focused vaccine design. It should be noted that this study prioritised the validation of rGn and rGc for IgM detection. Future studies will include IgG evaluation to provide a more comprehensive analysis of the functions of rGn and rGc.

In conclusion, this work demonstrates that prokaryotically expressed ectodomains (rGn, rGc) serve as effective antigens for acute-phase serodiagnosis. The results obtained in this study are of paramount importance, as they furnish vital immunogenicity data for these pivotal viral regions. Furthermore, they serve to deliver validated IgM-specific reagents and lay the foundation for the research on vaccines and therapeutic drugs based on DBV proteins.

## Data Availability

The original contributions presented in the study are included in the article/Supplementary material, further inquiries can be directed to the corresponding authors.
