# Supplementary figures and images for: Recombinant expression and immunogenicity verification of Dabie bandavirus proteins Gn and Gc
Source: Front Microbiol. 2025 Sep 5;16:1651194. doi: 10.3389/fmicb.2025.1651194 (PMC12446341; doi:10.3389/fmicb.2025.1651194)

Gn

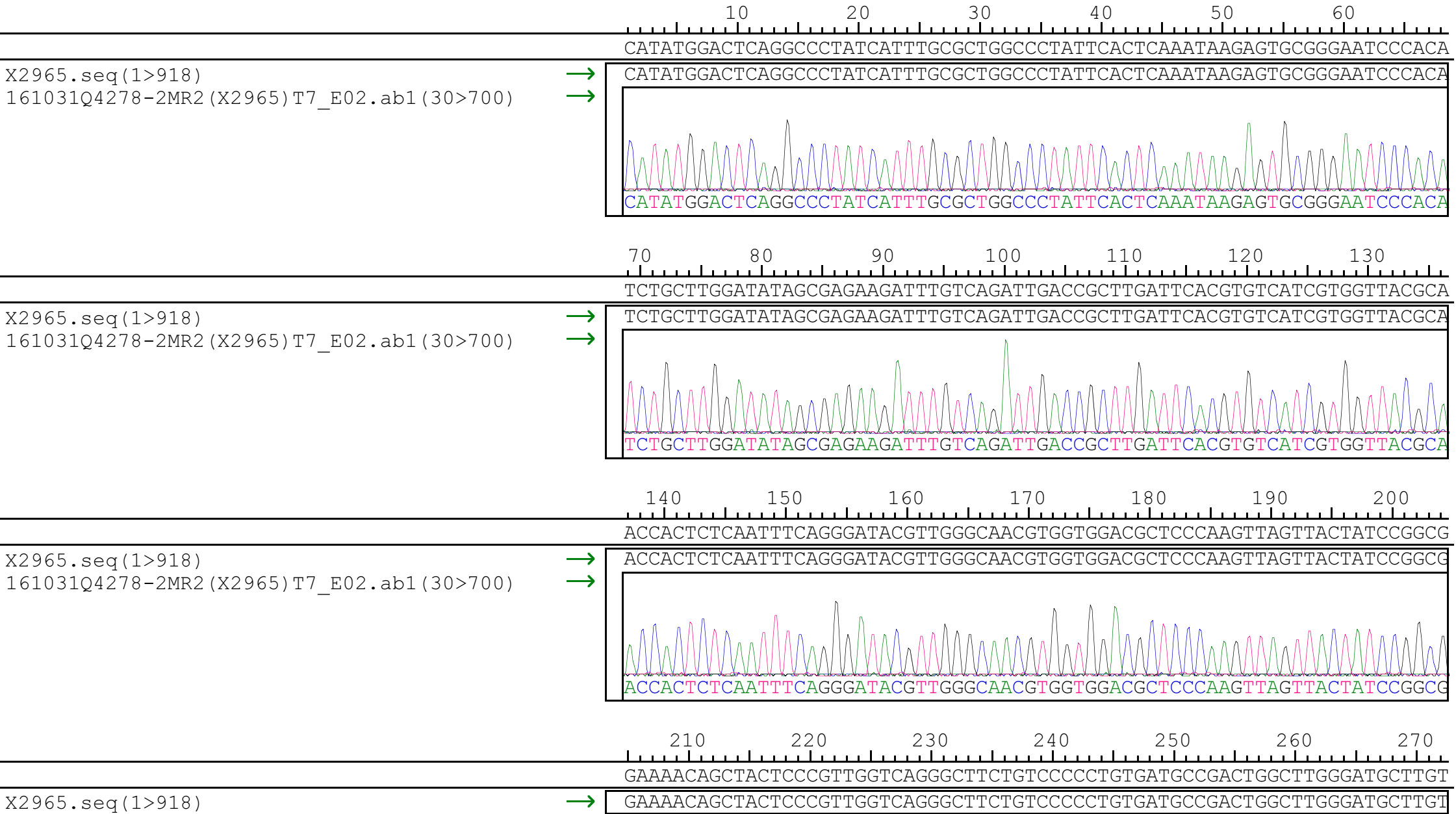

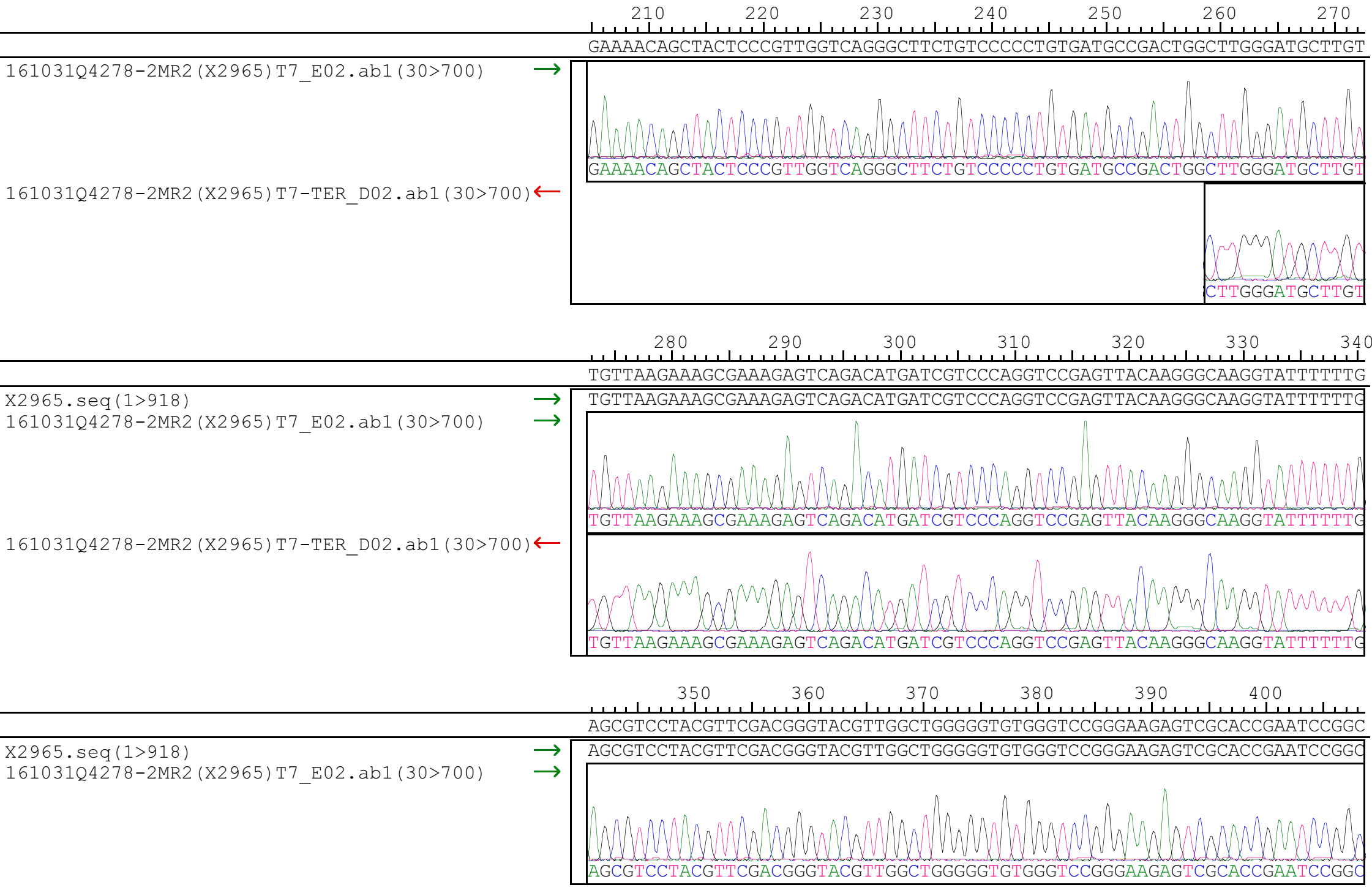

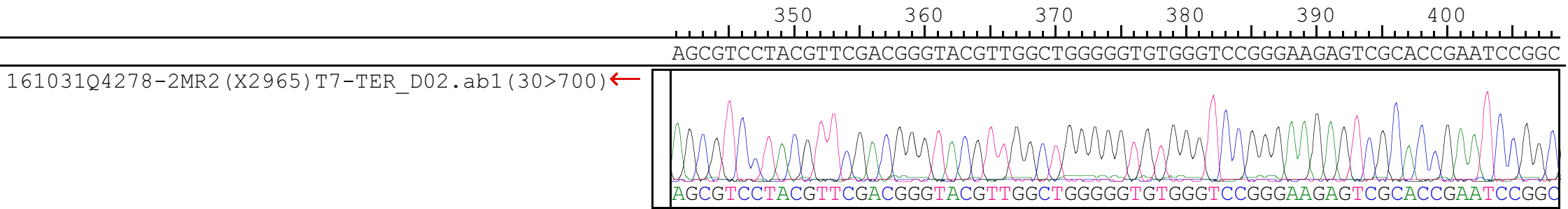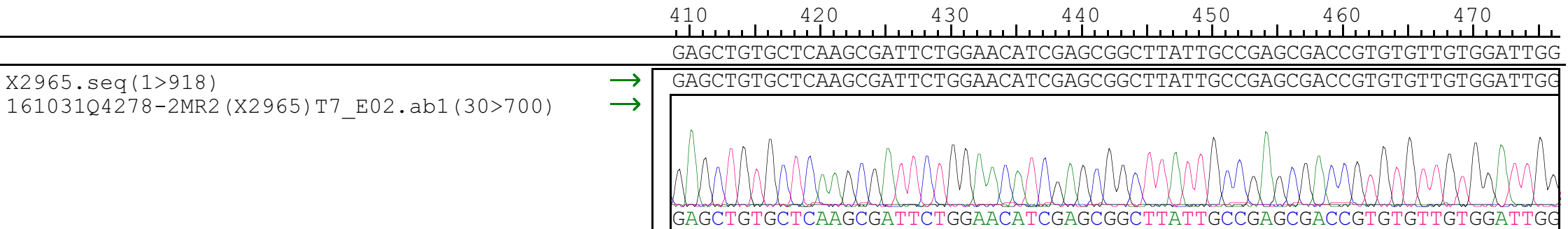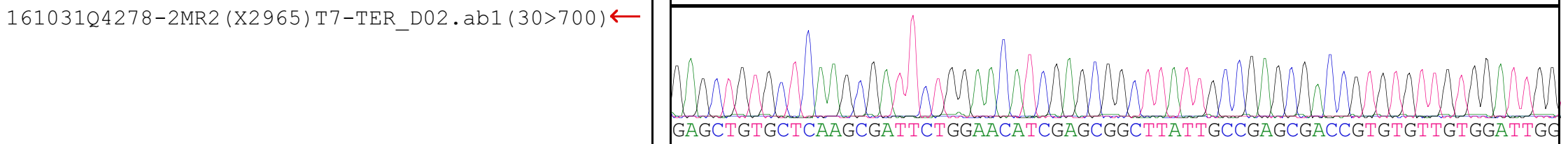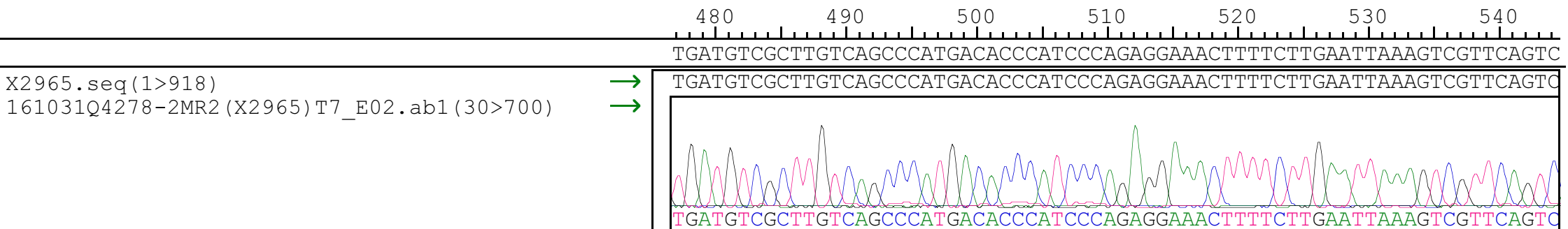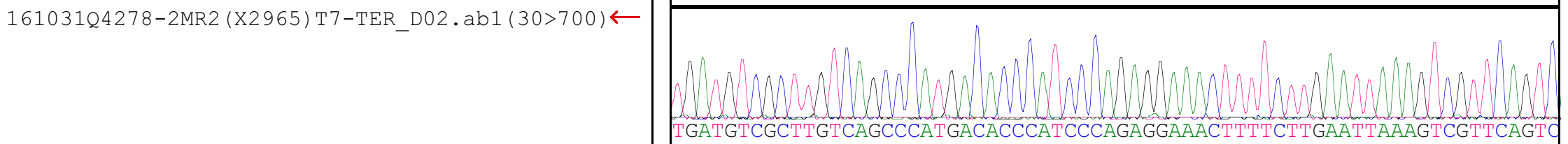

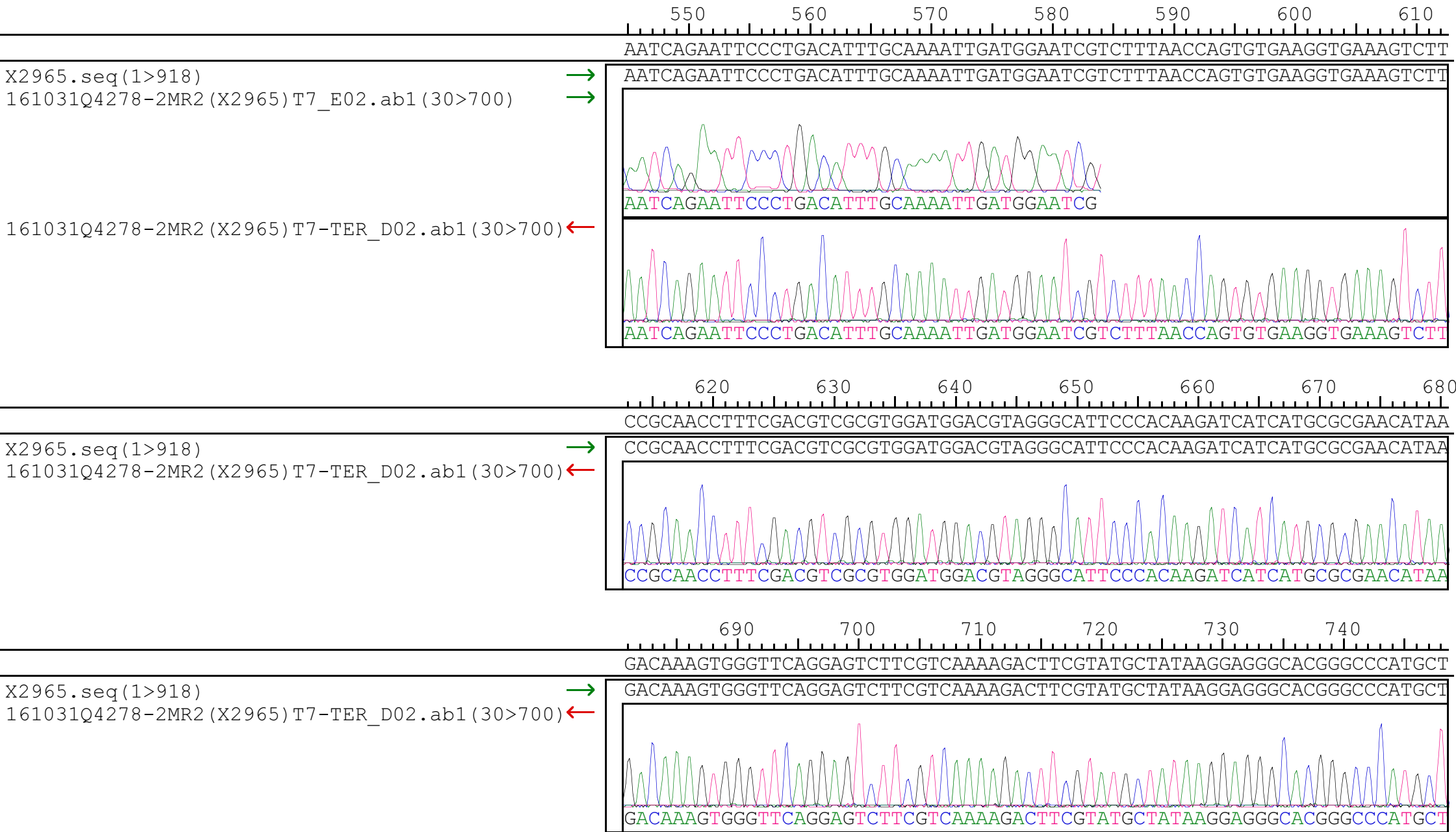

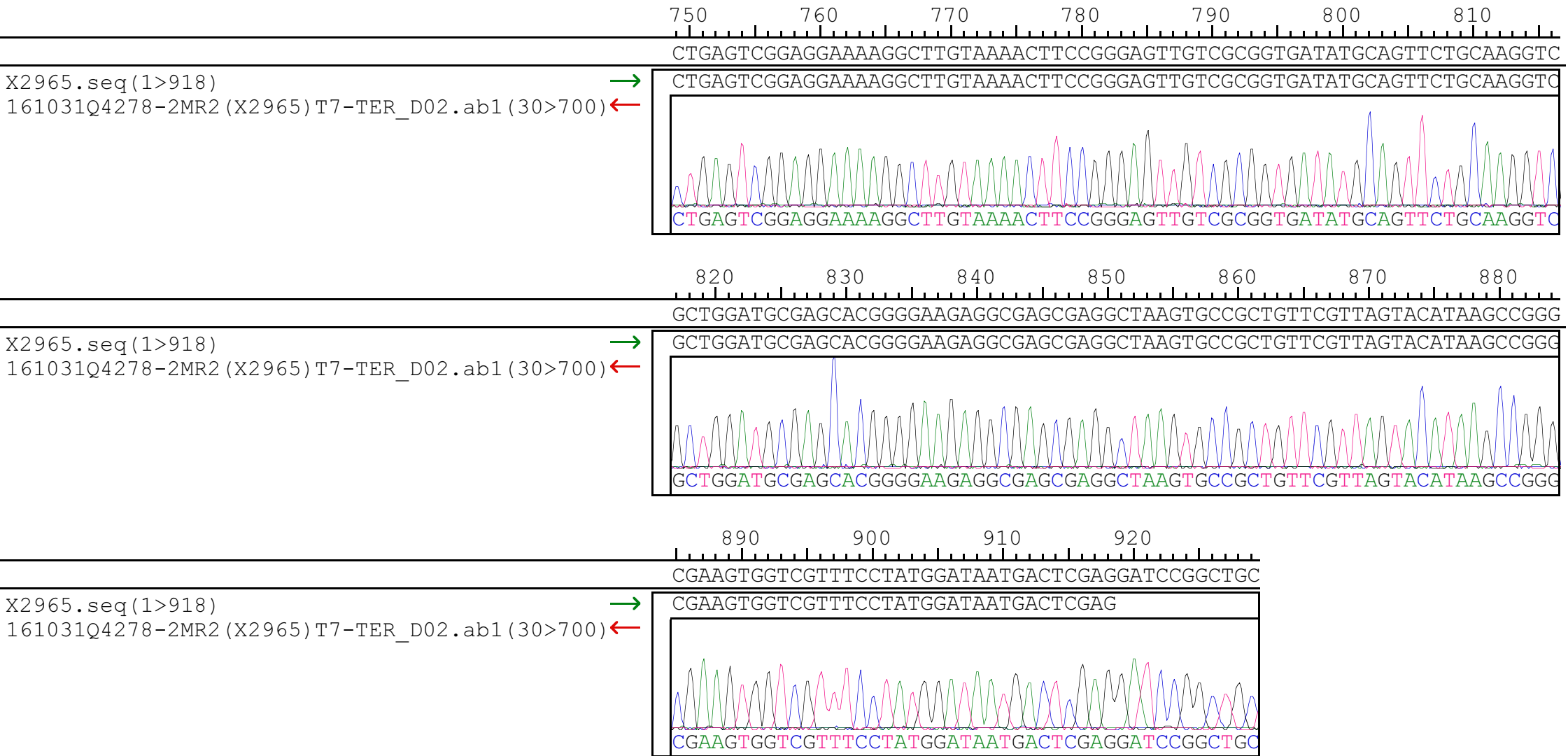

Gc

X2966.seq(1>1218)  
161031Q4279-1MR2 (X2966) T7\_B02.ab1 (30>700)

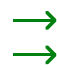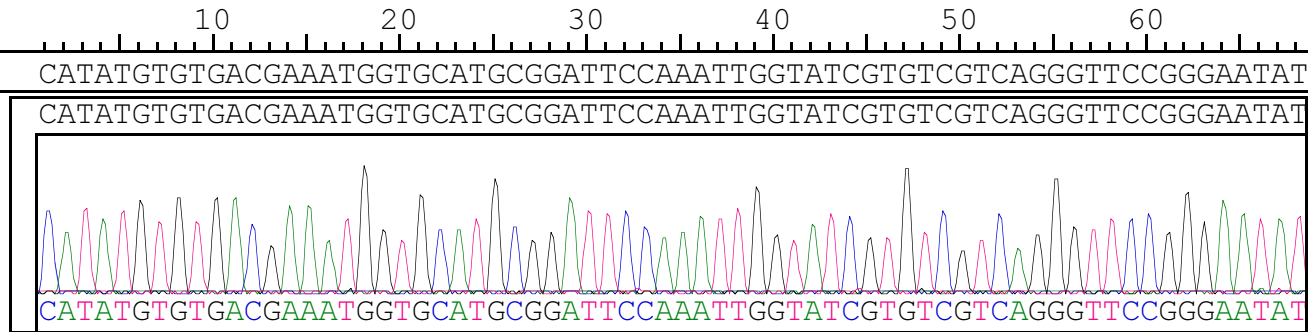

X2966.seq(1>1218)  
161031Q4279-1MR2 (X2966) T7\_B02.ab1 (30>700)

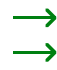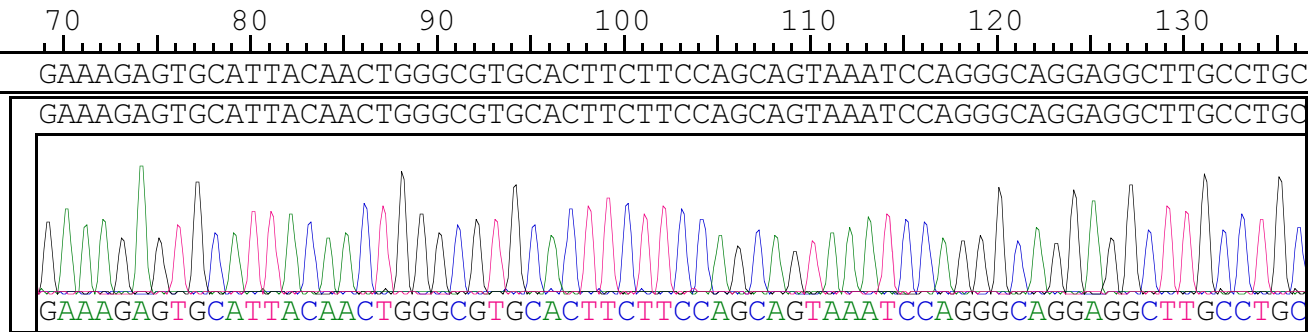

X2966.seq(1>1218)  
161031Q4279-1MR2 (X2966) T7\_B02.ab1 (30>700)

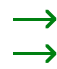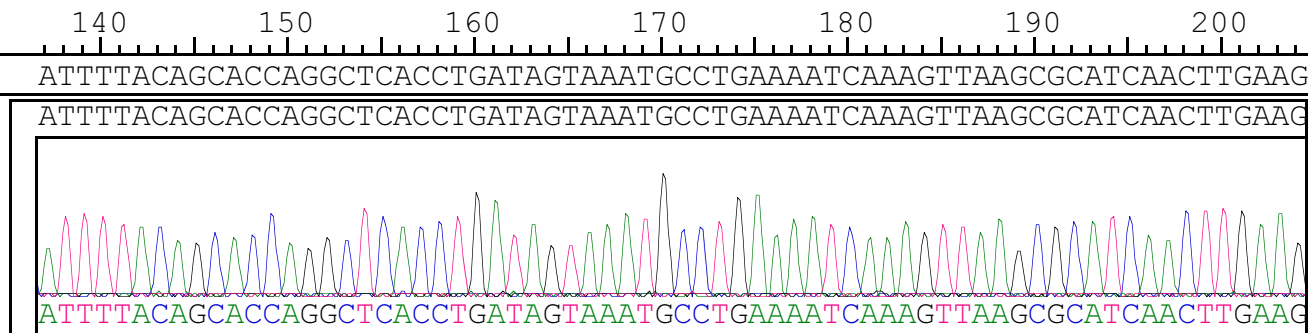

X2966.seq(1>1218)

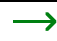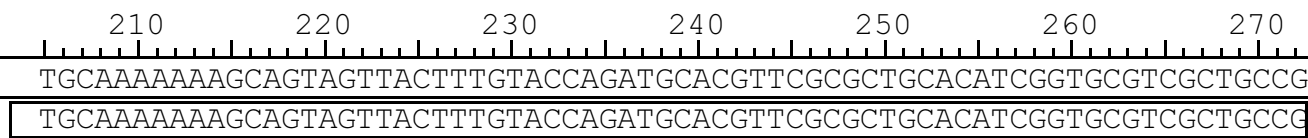

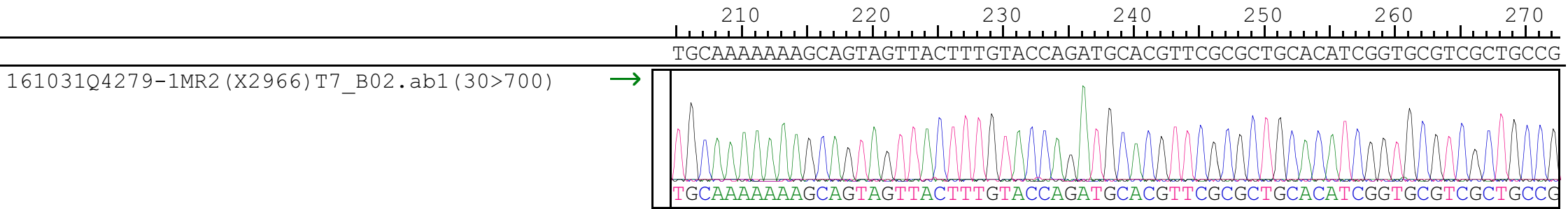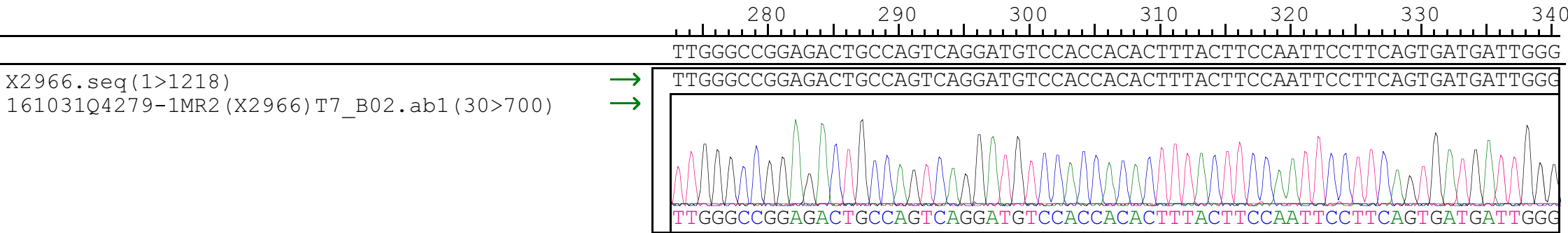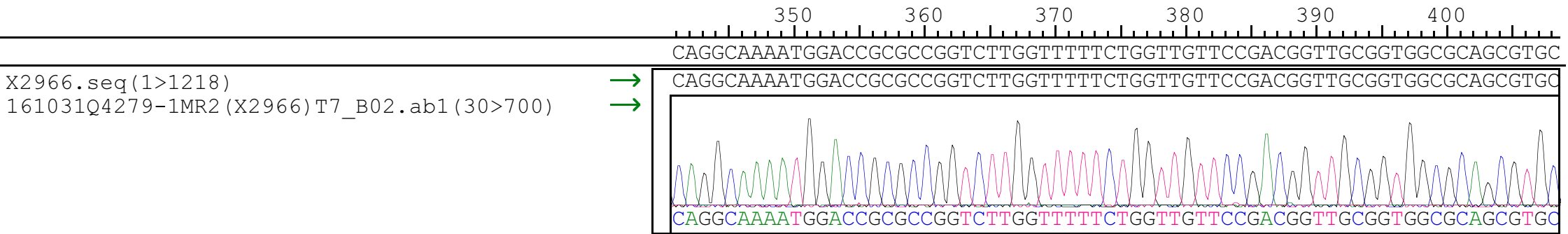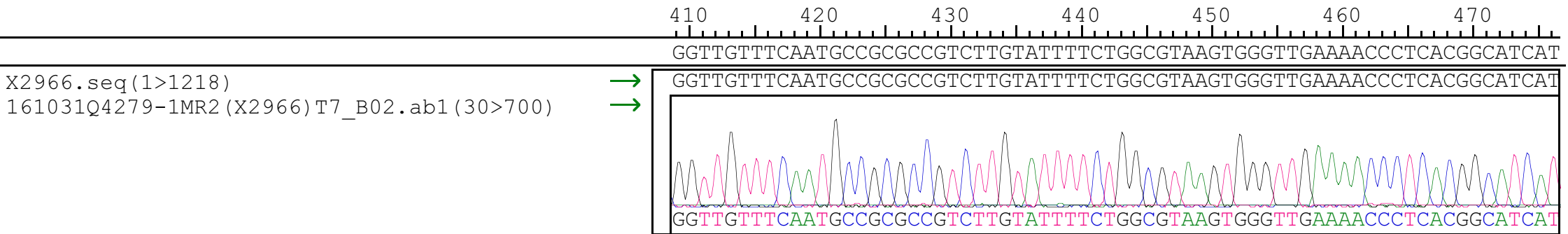

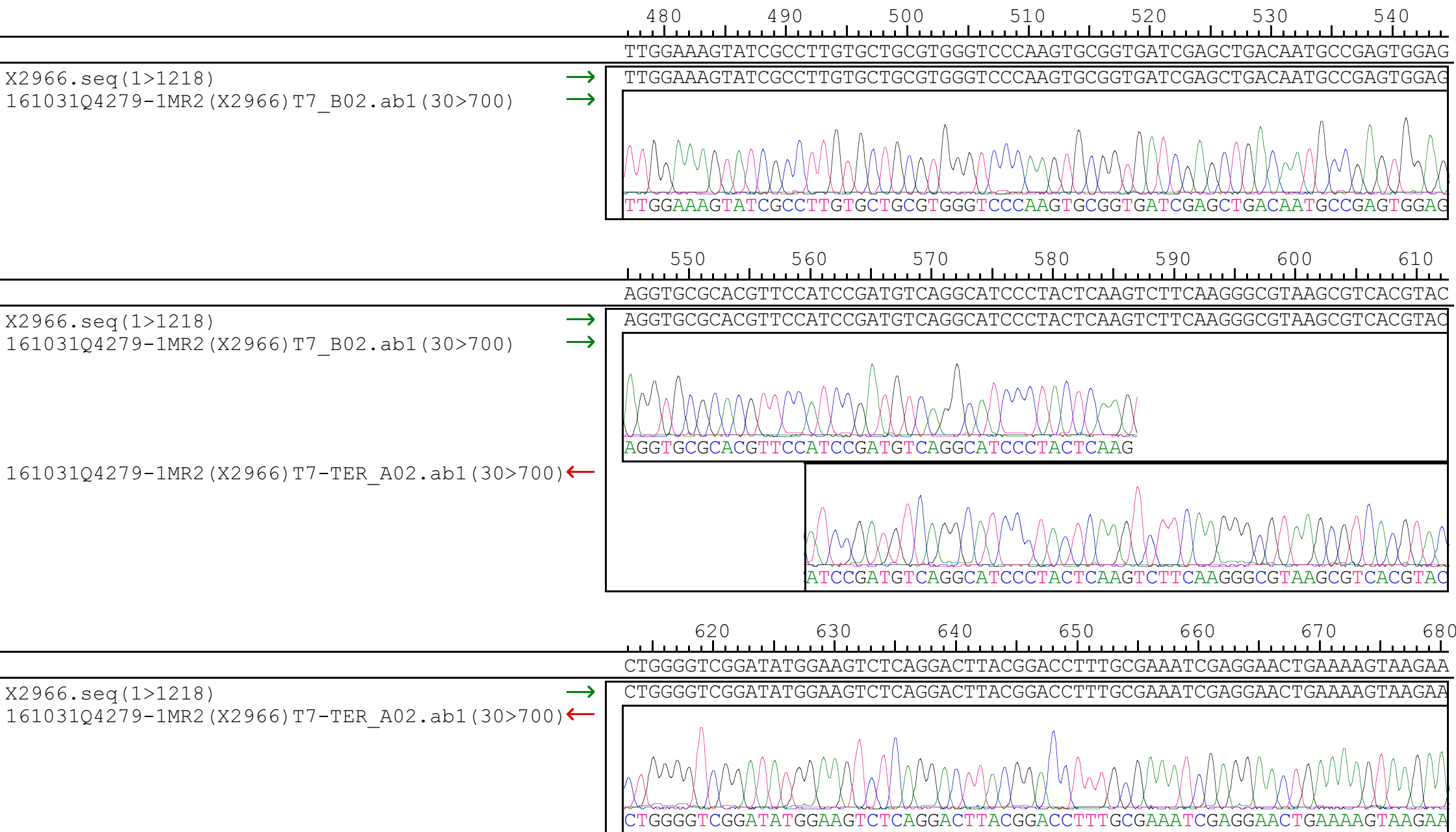

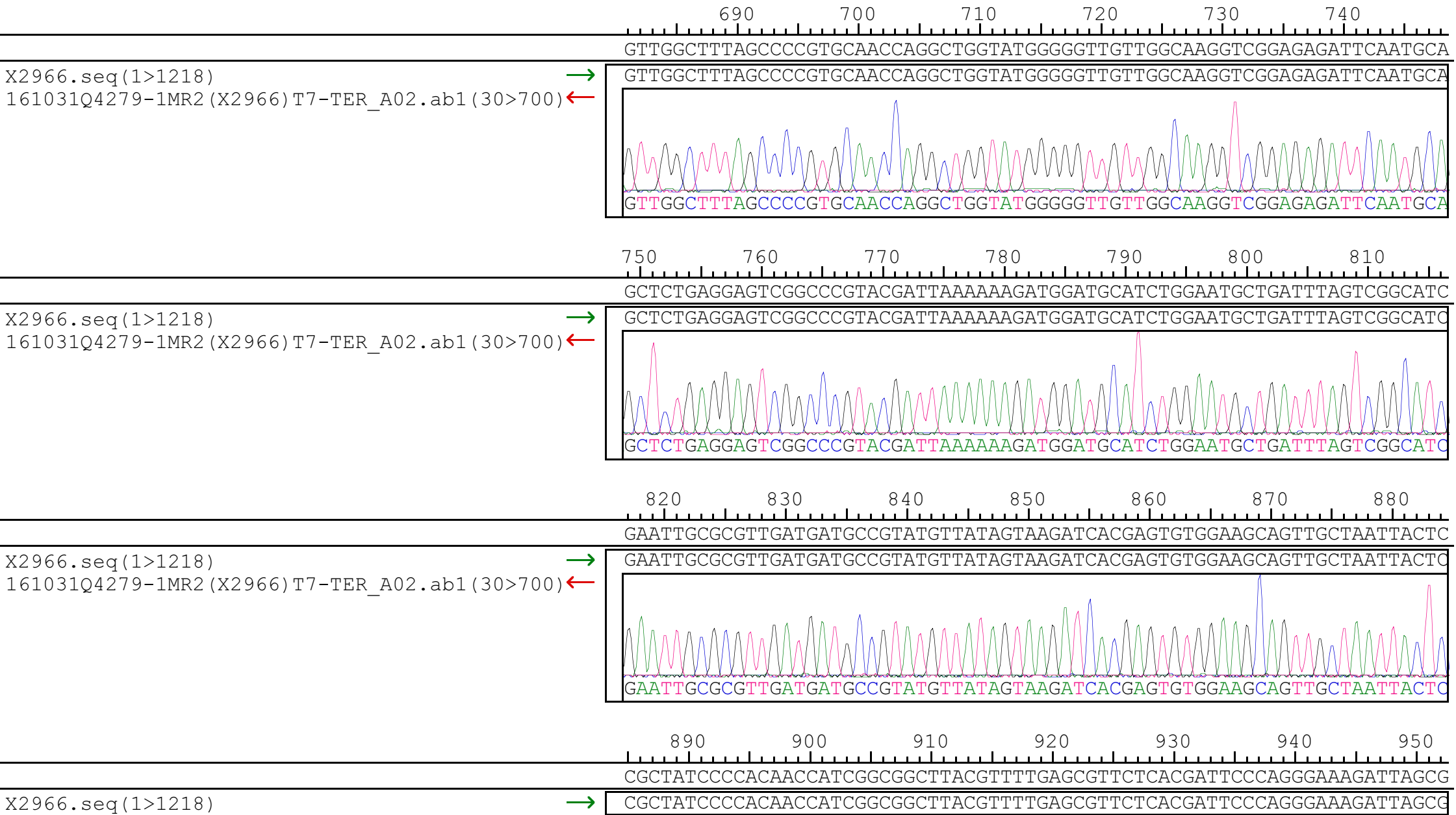

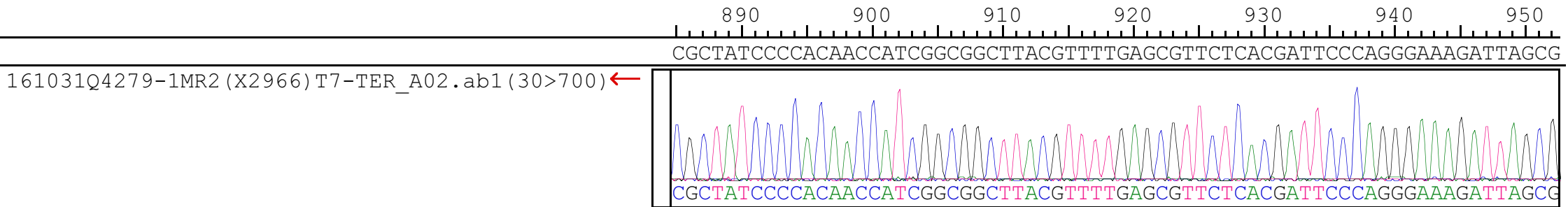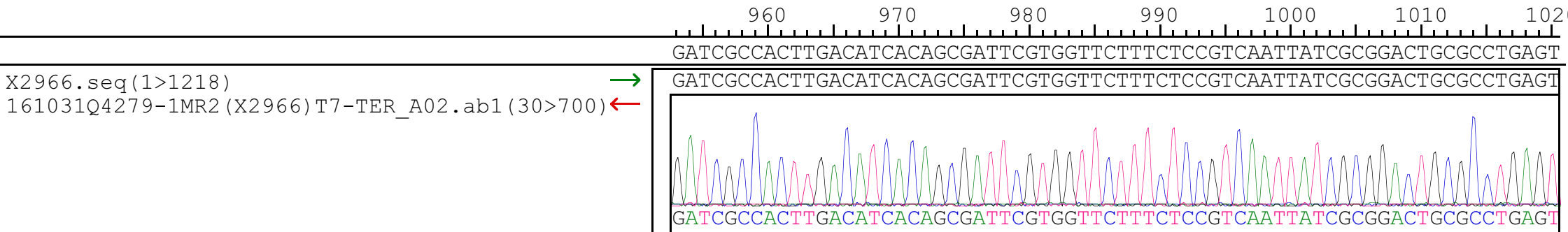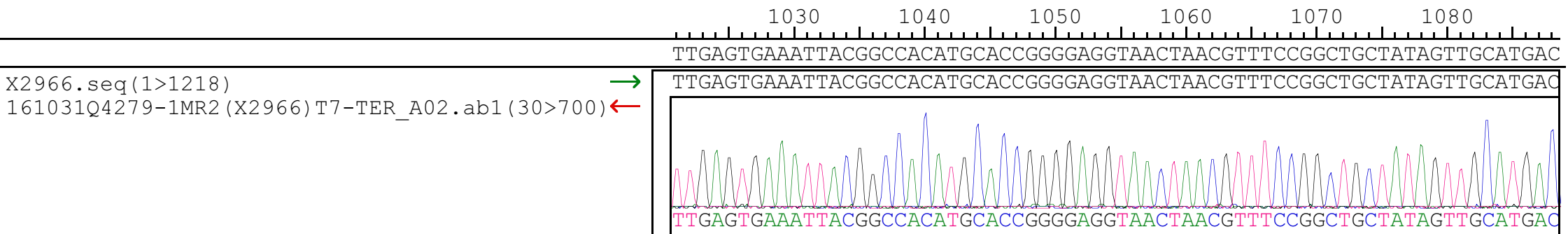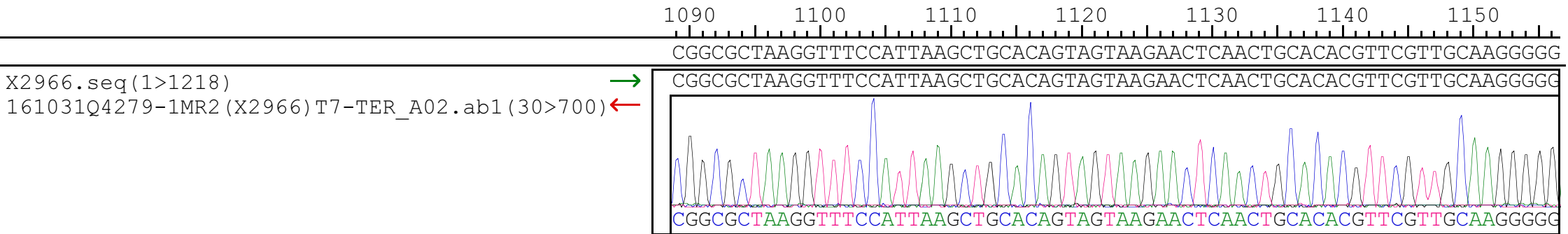

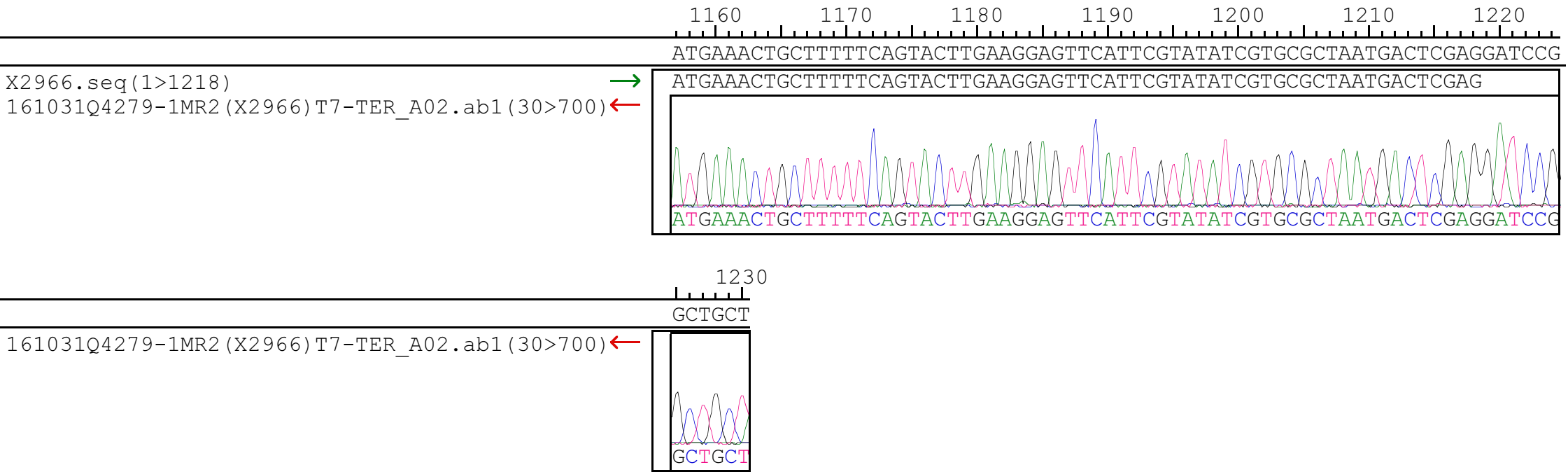

Supplement: Supplementary file 1 [file Data_Sheet_1.pdf]
